# A Dual-Branch Residual Network with Attention Mechanisms for Enhanced Classification of Vaginal Lesions in Colposcopic Images

**DOI:** 10.3390/bioengineering11121182

**Published:** 2024-11-22

**Authors:** Haima Yang, Yeye Song, Yuling Li, Zubei Hong, Jin Liu, Jun Li, Dawei Zhang, Le Fu, Jinyu Lu, Lihua Qiu

**Affiliations:** 1 School of Optical Electrical and Computer Engineering, University of Shanghai for Science and Technology, Shanghai 200093, China; 2 Key Laboratory of Space Active Opto-Electronics Technology, Chinese Academy of Sciences, Shanghai 200083, China; 3 Department of Obstetrics and Gynecology, Ren Ji Hospital, School of Medicine, Shanghai Jiao Tong University, Shanghai 200030, China; 4 Shanghai Key Laboratory of Gynecologic Oncology, Ren Ji Hospital, School of Medicine, Shanghai Jiao Tong University, Shanghai 200030, China; 5 Department of Obstetrics and Gynecology, Shanxi Bethune Hospital, Taiyuan 050081, China; 6 School of Electronic and Electrical Engineering, Shanghai University of Engineering Science, Shanghai 201620, China; 7 Shanghai First Maternity and Infant Hospital, School of Medicine, Tongji University, Shanghai 200092, China

**Keywords:** VAIN, colposcopic images, attention-guidance module, localization

## Abstract

Vaginal intraepithelial neoplasia (VAIN), linked to HPV infection, is a condition that is often overlooked during colposcopy, especially in the vaginal vault area, as clinicians tend to focus more on cervical lesions. This oversight can lead to missed or delayed diagnosis and treatment for patients with VAIN. Timely and accurate classification of VAIN plays a crucial role in the evaluation of vaginal lesions and the formulation of effective diagnostic approaches. The challenge is the high similarity between different classes and the low variability in the same class in colposcopic images, which can affect the accuracy, precision, and recall rates, depending on the image quality and the clinician’s experience. In this study, a dual-branch lesion-aware residual network (DLRNet), designed for small medical sample sizes, is introduced, which classifies vaginal lesions by examining the relationship between cervical and vaginal lesions. The DLRNet model includes four main components: a lesion localization module, a dual-branch classification module, an attention-guidance module, and a pretrained network module. The dual-branch classification module combines the original images with segmentation maps obtained from the lesion localization module using a pretrained ResNet network to fine-tune parameters at different levels, explore lesion-specific features from both global and local perspectives, and facilitate layered interactions. The feature guidance module focuses the local branch network on vaginal-specific features by using spatial and channel attention mechanisms. The final integration involves a shared feature extraction module and independent fully connected layers, which represent and merge the dual-branch inputs. The weighted fusion method effectively integrates multiple inputs, enhancing the discriminative and generalization capabilities of the model. Classification experiments on 1142 collected colposcopic images demonstrate that this method raises the existing classification levels, achieving the classification of VAIN into three lesion grades, thus providing a valuable tool for the early screening of vaginal diseases.

## 1. Introduction

Vaginal intraepithelial neoplasia (VAIN) is a group of lesions characterized by typical hyperplasia and carcinoma in situ in the vaginal squamous epithelium. It was initially identified during follow-up examinations after hysterectomy when vaginal lesions were detected. Currently, there are two main grading methods for VAIN. In one system, VAIN is classified into three grades: VAIN I, VAIN II, and VAIN III [1]. In a more recent classification system, VAIN I is defined as a low-grade squamous intraepithelial lesion (LSIL), whereas VAIN II and VAIN III are categorized as high-grade squamous intraepithelial lesions (HSILs) [2]. According to the latest WHO classification for tumors of the female reproductive tract (5th edition), vulvar squamous cell carcinoma and its precursor lesions are now categorized by etiology into HPV-independent and HPV-associated squamous types [3]. Due to the relatively low clinical focus on VAIN compared to cervical lesions, early statistics reported an annual incidence rate of VAIN of just 0.2–2 cases per 100,000, comprising only 0.4% of all lower genital tract intraepithelial neoplasias [4]. However, with advancements in screening techniques and increased clinical awareness of vaginal lesions, the incidence of VAIN has been rising steadily. Recent epidemiological data from Chinese studies indicate that VAIN now accounts for 23.7% of lower genital tract intraepithelial lesions [5], with an upward trend. Approximately 12% of high-grade VAINs have been shown to have the risk of developing into invasive vaginal wall cancer [6]. Despite extensive research on cervical intraepithelial lesions and well-defined diagnostic and treatment protocols, there is limited research on VAIN, and there is a lack of standardized diagnostic and treatment guidelines. Therefore, VAIN should receive more attention.

**Figure 1:**
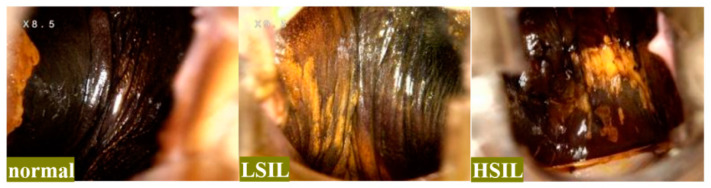
Three states of vaginal epithelium under iodine staining.

**Figure 2:**
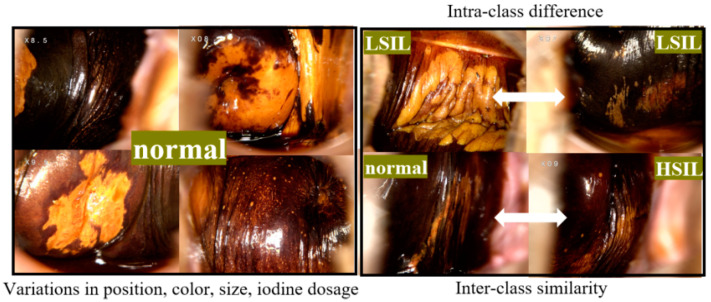
(**Left**): Variability in characteristics in the same type of vaginal epithelial lesion. (**Right**): Similar characteristics across different lesion types.

Colposcopy requires adequate illumination and local magnification to visualize the epithelium of the lower genital tract. The diagnosis typically involves observing changes in the cervical and vaginal epithelium after the application of acetic acid or iodine stains [7]. Figure 1 provides examples of different types of vaginal epithelial lesions under iodine stains. Variations in lighting, obstruction, angle, texture, and color in the affected areas can lead to significant variations in the same category, while different types of lesions may share similar features, as shown in Figure 2. As a result, the model struggles to account for all feature variations due to the limitations of the dataset. The overlap of features across categories can make decision boundaries obscure, making it more challenging to extract disease-specific features and increasing the overall difficulty of feature extraction.

**Figure 3:**
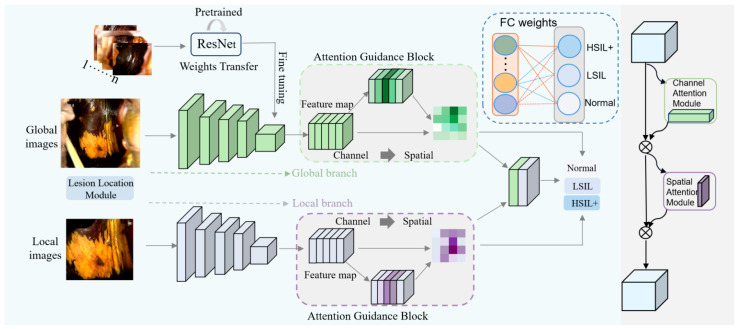
DLRNet is comprised of four main modules: the lesion localization and segmentation module, the dual-branch classification module, the attention-guidance module, and the weighted fusion module.

In this study, we developed a novel deep neural network architecture (DLRNet) to grade colposcopic impressions of VAIN. This method can improve existing classification levels and provide a valuable tool for the early screening of vaginal diseases. The framework of the model is shown in Figure 3.

The network was built on ResNet-34 architecture, which processes both global and local images as input. An attention-guidance module was developed to enhance feature interaction and optimize the benefits of both pathways [8]. The attention mechanism guides the network to prioritize critical areas, enabling it to extract pertinent disease features from these regions. The principal contributions of this study are summarized as follows:

1. DLRNet, a new lightweight dual-branch lesion-aware network model, has been proposed for small colposcopic datasets.
2. In this model, the different stages of vaginal lesions are classified, aiding doctors in reducing missed diagnosis rates and improving diagnostic accuracy. The network enhances classification performance by mutually learning global and local features and automatically integrating contextual lesion information.
3. Through spatial and channel attention mechanisms, the attention-guidance module learns disease-specific features, directing the model’s focus toward these particular characteristics. The optimal layer count for ResNet was determined through experimentation.
4. A clinical colposcopic image database comprising 1142 images from three different types of vaginal lesions was established. Each image in the database is accompanied by corresponding labels and segmentation annotations.

The experimental results indicated that the proposed model excels in both detecting and classifying vaginal-type images.

This paper is organized as follows: Section 2 provides a review of the related literature. In Section 3, the proposed DLRNet is introduced. Section 4 and Section 5 present the experimental results, along with their analysis. Section 6 offers a conclusion summarizing the key findings.

## 2. Related Work

The colposcopic impression of VAIN is variable and may present confusing image features depending on a number of factors, including the patient’s vaginal inflammation and hormone levels. Therefore, senior colposcopists always have a higher colposcopic accuracy to diagnose histologic VAIN [9]. However, there is a significant shortage of highly experienced colposcopists in rural areas of China, accompanied by lengthy waiting periods for colposcopy and biopsy pathology. Consequently, there is an urgent requirement for a real-time and precise diagnostic approach to assist colposcopists in their diagnostic and treatment procedures.

Research indicates that the severity of cervical lesions correlates with that of vaginal lesions. VAIN grading is positively associated with concurrent CIN (cervical intraepithelial neoplasia) grading. Furthermore, this consistency increases with age. The cervix, vagina, and vault share a similar environment, suggesting a possible common etiology for VAIN and CIN. This underscores the importance of examining the vagina when CIN is suspected. The clinical features of VAIN are similar to those of CIN, and the diagnosis and treatment principles for CIN are also applicable to VAIN. Artificial intelligence-based computer-aided diagnosis offers efficient, scientific, and accurate processing of clinical data, becoming an important branch of medical AI. Medical imaging and endoscopy have long been critical auxiliary diagnostic tools in clinical settings. Recent developments in convolutional neural networks (CNNs) for medical imaging have shifted the focus toward classification techniques based on deep learning (DL). However, in these DL methods, decisions are often made based on a single complete global image, overlooking a more detailed examination of lesion areas, which may contain additional physiological information. Moreover, due to the scarcity of colposcopic image data, transfer learning techniques are particularly crucial, as they leverage pretrained model parameters [10], providing an effective starting point for training new models and overcoming challenges related to limited data availability.

There are still challenges in the classification of actual nonterminal colposcopic images. First, traditional machine learning techniques often have variability across different datasets. Second, CNNs, which operate as end-to-end models without requiring manual feature extraction, are prone to overfitting when they are applied to small vaginal datasets [11]. Third, the collection of large-scale cervical colposcopic image datasets is challenging, and small sample sizes can easily cause the model to learn noise in the data rather than the underlying data distribution, resulting in poor generalization ability on new data. Based on current research findings, relevant technologies still have significant limitations in clinical application [12]. Researchers predominantly make decisions based on a single complete colposcopic image, overlooking the need for a more detailed examination of lesion areas that provide additional physiological information. Therefore, achieving an automated diagnosis of the severity of VAIN remains challenging.

Multibranch learning involves training multiple models simultaneously to explore more information about the target, effectively improving learning efficiency and prediction accuracy [13]. Consequently, multibranch learning techniques are increasingly utilized for a range of medical image analysis tasks encompassing both segmentation and classification [14]. For instance, Dolz et al. [15] proposed a multimodal network for multiple sclerosis lesion segmentation by strengthening dense connections where CNNs corresponding to each imaging modality were interconnected at each layer. Recent multimodal approaches have achieved high accuracy in breast cancer classification by integrating information from SWE or color Doppler images with B-mode images [16]. Additionally, Wang et al. [17] developed a dual-branch network that automates the classification and segmentation of esophageal lesions, integrating contextual information from two perspectives and extracting fine-grained features for precise lesion classification. Therefore, this paper proposes a dual-branch framework that combines original images with lesion segmentation maps using both global images and localized lesion regions to classify VAIN and enhance target differentiation.

Multibranch models combine channel and spatial attention mechanisms to emphasize critical features in the target region while reducing the influence of irrelevant data and enhancing both the network’s performance and its capacity to detect key features. Recent research has concentrated on incorporating attention mechanisms into deep learning models using straightforward plugin modules that create weight masks to enhance task-specific information [18]. Hu et al. [19] introduced the squeeze-and-excitation module, which uses global average pooling to model nonlinear cross-channel interactions, followed by two fully connected layers and a sigmoid function to assign channel-specific weights. As well as the channel attention mechanism, Woo et al. [20] proposed an efficient attention module that sequentially integrates channel and spatial attention mechanisms, forming a hybrid approach for dynamic feature refinement. This study presents an enhanced attention strategy that integrates both spatial and channel attention mechanisms [21]. The final layer includes several pooling operations and multilayer perceptrons to enhance feature mapping from both global and local branches, thereby improving contextual information integration.

## 3. Proposed Method

### 3.1. Local Lesion Segmentation Module

**Figure 4:**
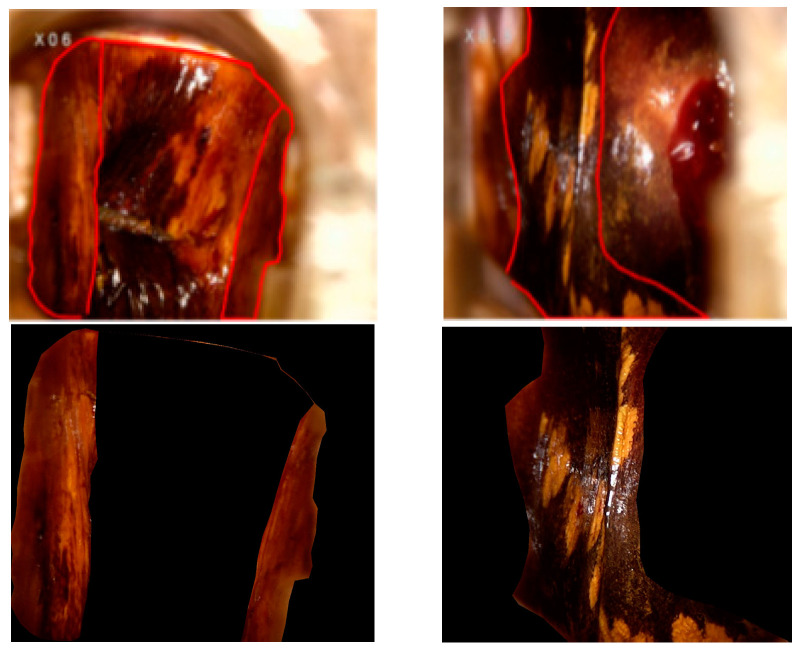
Localized colposcopic image: The red contours indicate the key area of interest in the vagina. The images above show the original with a red boundary, and the images below show the segmented results.

The localization features provided by the vaginal segmentation map are crucial for classification, but have not been fully exploited. Lesions from different categories may present similar appearances. Local images offer more distinguishing texture and color information than global information. Thus, combining global and local perspectives is essential for the effective diagnosis of the severity of VAIN. Local feature maps annotated for lesion areas in colposcopic images using Lableme software are utilized. The version of the software we are using is LabelMe Windows version 5.4.1. Pathological results serve as the gold standard for annotation, ensuring that each original image corresponds accurately. As shown in Figure 4, the global image confirms the colposcopic environment, while the local image reveals additional local and detailed features. Only the key area in the red boundary is retained in the segmented images. The remaining parts are masked in black.

### 3.2. Two-Branch Classification Module

The two-branch network takes both the full colposcopic image and the localized lesion area as inputs, generating an end-to-end classification result as the output. As shown in Figure 3, the two-branch network utilizes the ResNet-34 model as its feature extraction backbone. The global path extracts features from the full colposcopy image, while the local path focuses on lesion-specific features from the pathology region. In the output layer, the depth features from both branches are mapped into a unified representation space. Additionally, an attention module is positioned before the feature cascade to extract finer discriminative features and highlight the contextual information of the lesion region, enhancing classification performance. The integrated feature representation is then forwarded to the decision layer to predict the colposcopic intraepithelial neoplasia status. In contrast, multitask learning utilizes domain-specific insights from related tasks to promote inductive knowledge transfer, improving both prediction accuracy and generalization for each individual task [22]. We proposed a multi-input fusion method that utilizes a two-branch ResNet model to enhance performance. The model has two input branches, each of which undergoes a shared model for feature extraction and representation learning. The output of each input branch is further processed by an independent fully connected layer to obtain two independent feature representations. The weights are adjusted based on the problem’s characteristics and the data’s relevance. The final feature representation is obtained through weighted averaging, enabling the model to simultaneously learn both global and local features, thus overcoming challenges related to varying lesion locations. This approach significantly enhances the classification performance and generalization ability of the model, ensuring robustness in various applications.

### 3.3. Attention-Guidance Module

Attentional mechanisms, inspired by human vision, allow neural networks to focus on specific features and select relevant inputs $F_{in}\in R^{C\text{×}H\text{×}W}$, In this context, ‘C’ represents the number of channels, ‘H’ the height, and ‘W’ the width of the input feature map. The channel attention module emphasizes the interdependencies between the deepth dimensions of the channel graph, thus improving the feature representation of a particular semantic meaning. The spatial attention module then captures the relationships between feature points, integrating a wider context into the local features to enhance their representation. F with subscript i represents the i-th input. F with superscript k denotes the k-th feature. F with both superscript and subscript indicates the layer-specific feature. Specifically, the feature maps are first compressed through average and max pooling to gather the spatial information from the feature maps $F_{in}$. The aggregated features $F_{\max}^{c}\in R^{C}$ and $F_{\mathrm{avg}}^{c}\in R^{C}$ are then fed into a multilayer perceptron (MLP) consisting of $F_{c1}$ layers, a Relu activation function, and $F_{c2}$ layers, generating the following Formula (1) [23]:

(1) Ac=σ[W1Relu(W0Fmaxc)+W1Relu(W0Favgc)]

In this context, $W_{0}$ and $W_{1}$ denote the weights of the MLP, while the Sigmoid function is applied to the normalized attention weights. The parameters $W_{0}$ and $W_{1}$ represent the weights of the multilayer perceptron (MLP). Subsequently, the Sigmoid function is applied to the normalized attention weights, transforming them into a range between 0 and 1. The S-type function *σ* applies the attention weights to the original high-level feature maps, generating the channel attention feature maps $F_{c}=R^{C\text{×}H\text{×}W}$:

(2) $$F_{c}=F_{in}⊗A_{c}$$

(3) $$\sigma (x)=\frac{1}{1-e^{-x}}$$

where ⊗ denotes element-wise multiplication, facilitating the propagation of attention weights across the spatial dimension.

To improve feature discrimination across spatial domains, this study utilizes a spatial attention model that incorporates key contextual information into local features, enhancing their capacity to distinguish between different lesions [24]. The features are combined along the channel axis through maximum and minimum pooling operations applied to the channel attention features, producing spatial feature mappings $F_{\max}^{s}\in R^{H\times W}$ and $F_{\mathrm{avg}}^{s}\in R^{H\times W}$. Specifically, this process involves using convolutional operations to transform local features from the input image into mappings that have spatial relevance in the feature space. This spatial feature mapping helps the network recognize and understand different lesion characteristics in specific areas, thereby enhancing classification accuracy. These two feature mappings are then projected into the joint space, and the 2D spatial attention mapping is learned by another convolutional network.

(4) $$A_{c}=\sigma (Conv(Concat(F_{\max}^{s},F_{\mathrm{avg}}^{s})))$$

In this context, *σ* denotes the Sigmoid function used to normalize the attention weights, *Concat* indicates the concatenation operation for merging features, and *Conv* refers to the convolutional network consisting of flattened and fully connected layers [25]. Finally, the channel attention feature map *F*_*out*_ and the spatial attention feature map *A*_*s*_ are multiplied to obtain the lesion guidance feature map *F*_*out*_:

(5) $$F_{out}=F_{c}⊗A_{s}$$

In this regard, the attention-guidance module first introduces global and local branches, followed by the concatenation of the high-level semantic features extracted from both. In this way, by emphasizing lesion-related features across the whole image and within local abnormal regions, irrelevant areas are minimized, enabling a more focused attention on the semantic context of the lesion. The attention-guidance module enhances the model’s ability to prioritize disease-relevant features while eliminating irrelevant ones. In addition, it is incorporated before the feature fusion step between the two branches, enhancing the consistency of attention features and improving classification accuracy.

**Figure 5:**
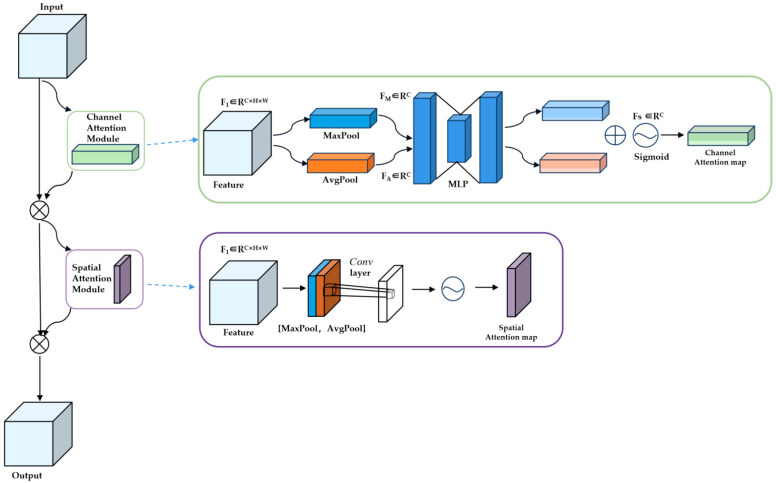
Network architecture of attention-guided blocks.

The input is derived from the features extracted by the backbone network. As illustrated in Figure 5, the channel attention module applies two spatial pooling methods and then sends the results to the frame network to merge the feature maps. After the sigmoid function is applied, the channel attention coefficients are multiplied by the input features. The spatial attention block combines features at the channel level using two methods. The spatial attention coefficients are obtained through convolution and the sigmoid function, and then multiplied by the original image to generate the final feature map.

### 3.4. Pretrained Network Module

Fine-tuned pretrained networks are widely applied in deep learning for the analysis of medical images with limited samples. The term transfer refers to the process of transferring neural network weights, convolutional kernel parameters, and bias values. In the same network model, pretrained weights from one dataset serve as the initial values when training on a new dataset. This enables small adjustments to the original weights based on the feature extraction requirements of the new task, improving its performance. This method reduces training time and effectively mitigates the issue of limited data. Due to the limited availability of training data, most medical image processing techniques rely on deep learning and transfer learning. Xu et al. [26] utilized a pretrained AlexNet model to classify cervical dysplasia in colposcopy images, demonstrating improved performance over conventional machine learning approaches in the classification of CIN 2+ lesions. Sato et al. [27] used deep learning to classify lesions in colposcopic images.Despite suboptimal results, their research highlighted deep learning’s potential for classifying colposcopy images. In particular, in the study [28], they proposed a multimodal deep network for diagnosing cervical dysplasia, setting a new benchmark in visit-level classification. Tan et al. [29] proposed sequential fine tuning, a new transfer learning method, to diagnose lung diseases, including cancer and tuberculosis (TB), using bronchoscopy images. Yuan et al. [30] enhanced a deep learning model with rotational invariance and image similarity constraints, leading to significant improvements in polyp detection from endoscopic images. Xiao et al. [31] utilized a transfer residual network (Re3sNet) for MRI-based brain disease detection, attaining leading performance in a multiclass classification task. Building upon the success of these studies, this research seeks to fine-tune all layer parameters using a pretrained ResNet residual network.

ResNet utilizes residual blocks that link layers to prevent gradient vanishing and to enhance learning efficiency. Each block consists of a convolutional layer and a residual connection. The output $a_{j}^{l}$ of the j-th unit of the convolutional layer is computed as follows:

(6) $$a_{j}^{l}=f\left(\sum_{i\in M_{j}^{l}}a_{i}^{l-1}*k_{i.j}^{l}+b_{j}^{l}\right)$$

where $M_{j}^{l}$ denotes the selected set of input feature mappings, $k_{i.j}^{l}$ denotes the learnable convolutional kernel, and f denotes the activation function. The convolution kernel $k_{i.j}^{l}$ acts as a sliding window, advancing by a fixed step size.

(7) H(x)=Y(x)+x

In this context, the variable x refers to the input provided to the structure, while Y signifies the sequence of convolution operations applied to this input. This notation emphasizes the functional relationship between the input and the subsequent processing steps involved in the convolutional layers. The Relu operation is performed before each weight layer in each residual block. Relu is the activation function, as shown below:

(8) Relu(x)=max(x,0)

After passing through multiple convolution layers, small images may lose edge information. To preserve all information, padding is applied to the input. The amount of padding is determined by the size of the convolution kernel. The padding size, denoted as P, applied to the input during convolution operations is determined by the size of the convolution kernel. This padding ensures that the spatial dimensions of the input image are preserved after convolution, thereby retaining important edge information that might otherwise be lost during the operation. By applying this padding, the network can effectively handle images with small dimensions and prevent the omission of critical features located at the borders [32]. For a convolutional kernel with edge length, the input image is the same size as the output when P=[k/2]. ResNet usually incorporates a single pooling layer, located after the final residual block. This layer filters the features from the convolutional layer, which helps reduce the number of training parameters and minimizes the risk of overfitting [33]. The output of pooling layer l is represented as follows:

(9) $$a_{j}^{l}=down(a_{j}^{l-1}M^{l})$$

where $M^{l}$ denotes the size of the l th pooling layer and down() is a downsampling function, which can be mean pooling, maximum pooling, or average pooling, depending on the specific pooling operation used. These pooling operations perform feature aggregation by selecting the most appropriate feature values within a sliding window and reduce the size of the output feature map to a multiple of three. As a result, the pooling layer reduces feature dimensionality, speeds up the training process, and mitigates the risk of overfitting.

The “FC” (fully connected) layer is a crucial component of many neural network architectures. In this layer, each neuron is connected to every neuron in the previous layer, allowing the model to combine complex features. It is responsible for mapping the high-level features extracted by convolutional layers to the final output classes, enabling accurate predictions. Typically, the FC layer is positioned toward the end of the network, serving as the final step in feature aggregation before producing the output [34]. The final stage of the ResNet model consists of multiple multi-neuron fully connected (FC) layers. These layers perform the two key functions of weighting and summing the extracted features. The FC layers fulfill three main roles: (i) combining features learned from the convolutional layers and aligning them with the label space; (ii) vectorizing high-dimensional, multichannel features extracted by the CNN into one-dimensional vectors; and (iii) serving as a classifier to consolidate all the knowledge learned so far. The multi-neuron FC layers can approximate complex nonlinear transformations and also fine-tune the CNN, enhancing its resilience to noise and disturbances.

In this study, a pretrained ResNet was first used as the initial weights of the colposcopic images, and the final FC layer was replaced with a new FC layer. The model has two input branches, each of which undergoes a shared ResNet model for feature extraction and representation learning. The output of each input branch was further processed by an independent fully connected layer to obtain two independent feature representations. In order to synthesize these two feature representations, a weighting strategy was used, where the weights can be adjusted according to the characteristics of the problem and the importance of the data. Ultimately, the result of weighting yields a comprehensive feature representation for subsequent tasks. This approach makes full use of the multi-input capability of the two-branch ResNet model at the algorithmic level and realizes the feature representation and fusion of the two inputs through a shared feature extraction module and an independent fully connected layer. Through weighted averaging, the information from multiple inputs can be effectively integrated to improve the performance and generalization ability of the model. Finally, the cross-entropy function is used as the loss function, which is represented as follows:

(10) $$Loss=\frac{1}{N}\sum_{i=1}^{N}L_{i}=-\frac{1}{N}\sum_{i=1}^{N}\sum_{c=1}^{M}y_{ic}\log(p_{ic})$$

where $p_{ic}$ shows the predicted probability that the ith observed sample belongs to category c. When M = 2, the task is two-category categorization, and when M > 2, the task is multicategory categorization; M denotes the number of categories to be categorized, and N the number of samples. The variable “i” represents the index of a sample, typically used to refer to the position of a specific data sample within the entire dataset. $y_{ic}$ is used to check whether y is equal to c. If the true category of the ith sample is equal to c, i will take the value 1. Otherwise, it will take the value 0.

## 4. Experiments

### 4.1. Dataset

We collected colposcopic image data from 1142 patients who underwent vaginal wall biopsy guided by colposcopy from June 2022 to June 2023 at Renji Hospital, affiliated to the Shanghai Jiaotong University School of Medicine, the International Peace Maternity & Child Health Hospital, and the Shanghai First Maternity and Infant Hospital. The imaging and biopsies were conducted by six experienced clinicians using Tongren TR6000C colposcopes and Leisegang (transliteration: Laiseikang) 3ML LED colposcopes. For each patient, one colposcopic image of the vaginal wall after a Lugol’s iodine test was collected. The dataset includes three types of images: HSIL, LSIL, and normal. Some examples are illustrated in Figure 1. To ensure patient privacy, the raw data were thoroughly anonymized upon extraction and converted into an anonymous file list.

**Figure 6:**
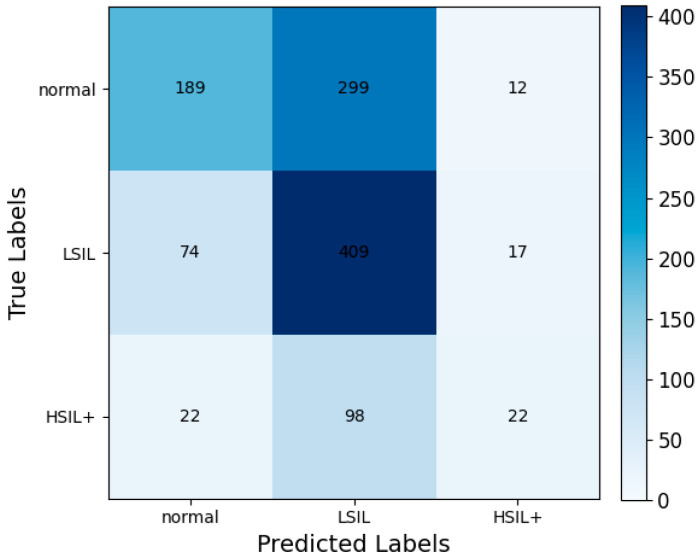
Confusion matrix of colposcopic predictions by physicians.

Two experienced experts initially screened the images, excluding those without corresponding pathological analysis or those taken from patients undergoing surgery or endoscopic removal. After thorough verification and quality control, the final dataset comprised 500 normal images, 500 vaginal LSIL images, and 142 vaginal HSIL+ images, as well as a segmentation dataset annotated using Lableme software for target detection training. The version of the software we are using is LabelMe Windows version 5.4.1. The dataset is evenly distributed. Clinical experts annotated each image category and corresponding mask label based on strict histological criteria. As shown in Table 1, images from the same patient were kept together to avoid overlap between the training, validation, and test sets. Moreover, due to the issue of data imbalance, which could negatively affect training outcomes, data augmentation was performed on the 142 vaginal images of the HSIL category. The original images were horizontally flipped, vertically flipped, and randomly flipped at angles ranging from 0° to 90°, as depicted in Figure 6. Ultimately, the 142 vaginal HSIL images were randomly augmented to 568 images. To improve training and testing efficiency, the resolution was reduced to 224 × 224.

The augmented dataset was split into training, validation, and test sets at a ratio of 7:1:2 for model training. The distribution of images is detailed in Table 1.

### 4.2. Experimental Setup

The program described in this study was trained on a platform equipped with an NVIDIA GeForce RTX 3080 Ti GPU with 30 GB of memory. The operating system of the experimental machine was Windows 10, and the proposed algorithm was built using PyTorch 1.7.0.

In the proposed method, a stochastic gradient descent (SGD) optimizer was employed. Other hyperparameters were set as follows: an initial learning rate of 1 × 10^−2^, adjusted using cosine annealing, a minimum learning rate of 1 × 10^−2^, adjusted using cosine annealing, a minimum learning rate of 1 × 10^−4^, a batch size of 32, and a total of 200 epochs. The multiclass cross-entropy was utilized as the loss function.

The proposed model in this study is referred to as DLRNet, and its development process involved multiple stages, totaling approximately nine weeks. The first phase was data preparation, which required about two weeks due to the need for extensive data cleaning and annotation. Following this, we spent one week modeling the design, which included selecting an appropriate architecture and tuning hyperparameters to optimize performance.

The training phase represents the highest time cost in the overall development process. Depending on the complexity of the model, we utilized high-performance GPUs to ensure that the training time for each model was kept in three to five days. For instance, training the ResNet-34 architecture, which serves as the baseline model, required four days on a dataset containing all images. Subsequently, we conducted a five-fold cross-validation to ensure the robustness of the model’s performance. Afterward, several weeks were spent making comparative analyses of the models and carrying out visualization experiments.

Upon completing the training, we spent an additional week on model validation and evaluation, ensuring that all performance metrics, including accuracy and ROC curve analysis, were thoroughly assessed [35]. Overall, the entire timeline from data preparation to final validation spanned approximately nine weeks, adhering to rigorous standards of model development.

### 4.3. Evaluation Metrics

To precisely quantify the model’s classification performance, the following metrics were introduced: accuracy, F1 score, precision, recall, and confusion matrix. Accuracy, calculated using Formula (11), measures the percentage of correctly classified instances, with values closer to 1 indicating better performance of the classification algorithm.

(11) $$Accuracy=\frac{TP+TN}{TP+TN+FP+FN}$$

Precision, as defined in Formula (12), measures the proportion of true positive predictions among all positive predictions. A value closer to 1 indicates better classifier performance.

(12) $$Precision=\frac{TP}{TP+FP}$$

Recall, as defined by Formula (13), quantifies the proportion of actual positives correctly identified by the model. Higher values, approaching 1, demonstrate the model’s effectiveness in identifying all relevant cases.

(13) $$Recall=\frac{TP}{TP+FN}$$

The F1 score, as defined by Formula (14), measures the balance between precision and recall. A higher value, approaching 1, indicates superior performance, demonstrating both accuracy and thoroughness in classification.

(14) $$F1=2\times \frac{Precision\times Recall}{Precision+Recall}=\frac{2TP}{2TP+FP+FN}$$

Furthermore, this study also employed standard evaluation metrics for assessing object detection performance, including true positives (TPs), false positives (FPs), and false negatives (FNs). TP refers to the correctly identified positive images, FP to the incorrectly detected instances, and FN to the positive samples misclassified as negative.

## 5. Results and Discussion

### 5.1. Selection of the Baseline Model

For the selection of a baseline model, choosing a deep learning network that aligns with the specific characteristics of the image classification task can significantly enhance the extraction of critical image features, thus improving classification accuracy. In this section, seven classic classification networks are employed to train on, validate, and test the raw endoscope image dataset [36]. For each network, the parameters achieving the highest accuracy on the validation set are used for testing on the test set. Finally, based on these results, the model with the best performance is selected as the baseline for processing vaginal image branches. Table 2 presents the classification results of the feature-encoding networks on the raw images.

These results are based on the benchmark model evaluation conducted using the raw endoscope image dataset listed in Table 1. As shown in the data from Table 2, ResNet-34 demonstrates relatively superior performance across the four metrics, achieving an accuracy of 0.7923, a precision of 0.7857, a recall of 0.5840, and an F1 score of 0.6700. In terms of accuracy, ResNet-34 outperforms the second-best network, ShuffleNetV2, by 0.0304. For precision, it exceeds MobileNetV2, ranked second, by 0.1019, and for the F1 score, it surpasses EfficientNet-B0, ranked second, by 0.0228. Although ResNet-34 does not achieve the highest recall, its overall performance across all metrics is superior to that of the other networks. Therefore, this study selected ResNet-34 as the benchmark model for handling the vaginal image classification task.

### 5.2. Ablation Study

In the classification test set of this study, colposcopists achieved an accuracy rate of only 54.29% when making correct judgments based on case information and colposcopic images combined with their experience. The biopsy detection rate was relatively low. As shown in Figure 6, most predictions made by colposcopists were concentrated in the LSIL category. While this approach reduces the probability of misdiagnosis, it also increases the number of biopsies [37], leading to a lower detection rate and prediction accuracy. These findings underscore the importance of developing a highly accurate automated VAIN classification model.

Ablation studies were conducted on the internal dataset to assess the impact of each submodule in the proposed method. The baseline model, built on the ResNet34 architecture with global images, served as the starting point, with each submodule added sequentially. The modules tested included single branch (Single), dual branch (Dual), pretraining (Pretraining), and attention guidance (Attention). All experiments were conducted using a consistent training setup, producing quantitative results. The DLRNet model outperformed single-branch models, which used either global or local images, highlighting the dual-branch approach’s effectiveness in combining features from both sources, as shown in Table 3.

**Figure 7:**
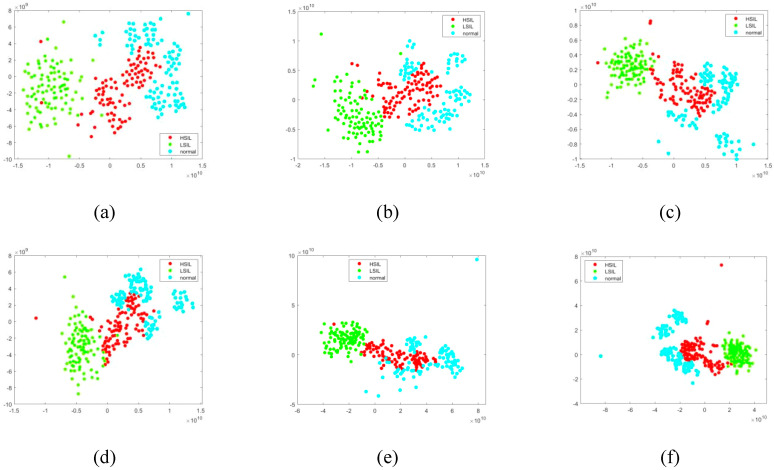
t-SNE visualization of ablation experimental results on the test set. (**a**) Single (global), (**b**) Single (local), (**c**) Dual (No pretrained), (**d**) Dual + Attention, (**e**) Dual* (Pretrained), (**f**) Dual* + Attention.

As depicted in Figure 7, the t-SNE visualization shows the results of the ablation experiments on the test set. It can be seen that the separation effect improves as each module is incorporated into the proposed framework. When comparing (c) and (e), features of the same type are more tightly grouped, indicating that the pretraining module aids in convergence and better models the feature distribution by capturing multivariable relationships [38]. A comparison between (e) and (f) reveals clearer distinctions between the normal, LSIL, and HSIL categories, confirming that the attention mechanism helps the network focus on disease-specific features.

As observed in the last row of Table 3, the introduction of the attention-guidance module enhanced the network’s ability to focus on regions of interest within the lesions. The right side of the table shows that, as modules are progressively added, performance metrics improve, particularly in distinguishing the more challenging vaginal LSIL and normal cases.

In order to assess the model’s effectiveness, the Wilcoxon signed-rank test was applied to compare the physician and DLRNet predictions. The results of this comparison are summarized in Table 4 below.

The results of the paired sample Wilcoxon test show that the median of the paired differences is 0.000, with a standard deviation of ±0.626. This indicates that the difference between the original and model predictions is, on average, minimal. The Z value is 6.275, and the *p* value is 0.000, suggesting that the difference between the original and model predictions is statistically significant [39]. Given that the *p* value is far smaller than the commonly used significance threshold of 0.05, it can be concluded that the difference between the model and original predictions is of practical significance. The Cohen’s d value is 0.989, indicating a large effect size. This suggests that the model has a significant impact on enhancing prediction accuracy and consistency, underlining its substantial contribution to improving prediction outcomes.

### 5.3. Comparative Experiments

To demonstrate the efficacy of the proposed method, comparative experiments were conducted, comparing 16 advanced classification techniques across three categories. These included the improved classical and transfer learning methods MobileNet-RVFL-CBA [40], ResNet-ELM-CBA [41], BN-AlexNet-ELM-CBA [42], and Deep transfer ResNet [43]. They also included the multibranch method ELNet [44], an esophageal dual-stream network that automatically integrates dual-view complementary lesion background information, extracting global and local features. Xie et al. [45] proposed a multibranch architecture for fundus disease classification, integrating a cross-attention module to enhance feature representation. The relevant code was sourced from open-source repositories and retrained on the dataset to produce classification results [46]. To maintain consistency, the same hyperparameters were used during the training process in this study.

To demonstrate the effectiveness of the proposed method, comparative experiments were conducted with State-of-the-Art classification techniques. A multimodal input classification network was utilized, and the dataset comprised global vaginal images and localized lesion images from this study.

Table 5 shows the comparison results between our method and existing advanced methods—the proposed DLRNet network achieved the highest accuracy. Compared to the classic classification methods mentioned, the DLRNet network emerged victorious, achieving higher accuracy than the methods in the classic networks. Among the existing methods that used vaginal images as the dataset, most focused on binary classification, and their accuracy did not meet the clinical diagnostic requirements [47]. The network proposed in this study fills the research gap regarding the classification of vaginal images, enabling a three-class classification that is more precise and significantly enhances accuracy.

**Figure 8:**
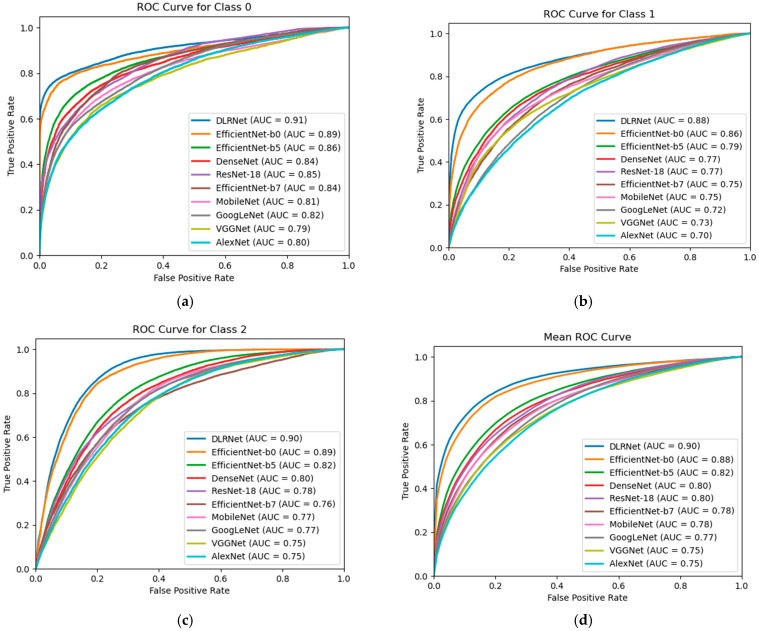
ROC curves for each disease, comparing classic models and the proposed method. (**a**) 0: Normal, (**b**) 1: LSIL, (**c**) 2: HSIL +, (**d**) Mean.

As shown in Figure 8, this study plotted the ROC curves for each category and compared them with classic methods. It is clear that the proposed method achieved the best classification performance across all categories.

The 16 models were included to compare the performance of our proposed model with other published medical image classification methods in terms of accuracy. In the field of medical imaging, it is essential not only to achieve superior accuracy compared to other approaches, but also to conduct a comparison of ROC curves. Following this, 10 models were selected based on the initial data comparison results to plot ROC curves for evaluating the models’ detection capabilities. Subsequently, eight models were chosen for further evaluation to compare their effectiveness in localizing lesion sites [48].

**Figure 9:**
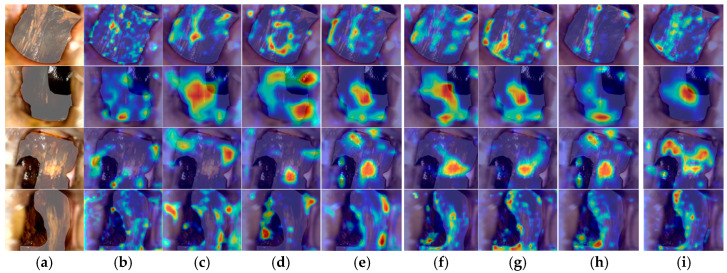
Grad-CAM visualizations comparing the proposed method with other classical methods: (**a**) Endoscopic images; (**b**) CNN; (**c**) VGG Net-D & Net-E; (**d**) Mobilenets; (**e**) ResNet (ILSVRC’15); (**f**) DenseNet-BC; (**g**) GoogLeNet; (**h**) EfficientNet; (**i**) DLRNet.

Table 5 presents the metrics of our method compared with other advanced methods for the collected dataset. The DLRNet method achieved the highest accuracy, precision, recall, and F1 score. Additionally, to validate the feature extraction capability of the designed method, Grad-CAM images were generated to demonstrate the proposed structure’s advantage in focusing on relevant features. The Grad-CAM visualizations, as shown in Figure 9, illustrate images of lesions under endoscopy. Compared to other methods, this framework effectively focuses on the disease lesions while suppressing irrelevant areas.

To explain why the DLRNet method outperforms other models in various metrics, we can analyze its strengths in feature extraction, contextual information integration, and the application of attention mechanisms.

Feature extraction: DLRNet employs a dual-branch structure that allows for simultaneous learning of global and local features. This capability enhances the model’s sensitivity to critical details in the images, enabling it to accurately identify complex lesions that may be challenging for other models. The integration of original images with segmentation maps further enriches the feature set, leading to improved classification performance.

Contextual information integration: The model effectively incorporates contextual information from both global and local perspectives. By leveraging multimodal data, DLRNet can better understand the spatial relationships within the images, which is essential for distinguishing between different lesion types. This comprehensive contextual analysis enables the model to make more informed predictions.

Attention mechanisms: The attention-guidance module of DLRNet plays a pivotal role in enhancing model performance. It directs the network’s focus toward relevant areas of the image while suppressing noise from irrelevant features. This targeted approach not only improves the model’s precision in identifying disease-relevant characteristics, but also helps maintain high recall rates by ensuring that significant features are not overlooked.

These factors collectively contribute to DLRNet’s superior performance across all evaluated metrics, establishing it as a reliable tool for vaginal image classification tasks. The ability to effectively extract and integrate features, coupled with a robust attention mechanism, underscores its potential for clinical application in gynecology and obstetrics. The DLRNet dual-branch architecture achieved superior accuracy, surpassing traditional methods. The per-class precision highlights its ability to effectively distinguish challenging samples. Unlike traditional image classification methods, our approach effectively captures local features and integrates global ones, enhancing the model’s ability to differentiate lesion types. In contrast to multibranch methods, ELNet can extract both global and local features. However, its performance is limited by its inability to focus on specific lesion details, primarily due to the lack of an attention mechanism. Comparing local and global inputs reveals that using images with mask-level local data generally enhances classification accuracy [49], emphasizing the crucial role of the local branch [50] in distinguishing diseases. These results validate the superiority and robustness of our approach, improving lesion detection in endoscopy and offering crucial assistance to physicians in diagnosing vaginal lesions.

## 6. Conclusions

This study presents a dual-branch lesion-aware network utilizing the DLRNet model for colposcopic lesion classification. The network simultaneously learns global and local features, seamlessly integrating contextual lesion information. The attention-guidance module focuses the network on prominent target areas, further exploring features associated with specific diseases. The dual-branch classification module integrates original images with segmentation maps from the lesion localization module, utilizing a pretrained ResNet residual network to fine-tune parameters at different levels, exploring disease-specific features from both global and local perspectives, and facilitating layered interactions. The feature guidance module directs the local branch network to focus on vaginal-specific features through spatial and channel attention mechanisms [51]. Ultimately, a shared feature extraction module and independent fully connected layers achieve the representation and fusion of features from the dual-branch inputs. The weighted fusion method effectively integrates multiple inputs, enhancing the model’s discriminative and generalization capabilities. Based on the data presented in Table 5, DLRNet demonstrates superiority across four evaluation metrics, with the following specific results:

DLRNet achieves an accuracy of 0.8572, a precision of 0.8557, a recall rate of 0.8513, and an F1 score of 0.8562. Compared to other models, DLRNet’s accuracy surpasses that of the second-best model, EfficientNet, by 0.0243, and its precision exceeds that of the second-ranked CLIP by 0.0092. Moreover, its recall exceeds that of the second-ranked CLIP by 0.0123, and its F1 score is 0.0213 higher than that of the second-ranking EfficientNet. On the whole, DLRNet’s overall performance across all other metrics is superior to that of other networks, demonstrating its effectiveness and reliability for this task. Ultimately, this will enable precise preoperative assessment and management of patients, reduce the incidence of surgical complications, and advance the practice of precision medicine in the fields of gynecology and obstetrics.

There are several limitations. First, this study is based on a database from several medical centers in Shanghai. To ensure its broad applicability in different regions and patient populations, a prospective study will be conducted to further validate the robustness and generalization ability of the DLRNet model in diverse clinical settings. Second, colposcopic images in combination with other investigations (e.g., HPV testing, histopathological analysis, etc.) can facilitate a more comprehensive assessment of the patient’s condition. In future studies, we will consider these multimodal data in the network to improve the accuracy and reliability of classification.

## Figures and Tables

**Table 1:** Detailed distribution of the colposcopic dataset.

	Training	Validation	Test	Total
Normal	350	50	100	500
LSIL	350	50	100	500
HSIL+	398	57	113	568

**Table 2:** Classification results of the original image feature-encoding network.

Model	Accuracy	Precision	Recall	F1-Score
VGG-16	0.6579	0.6766	0.5257	0.5916
ResNet-18	0.6385	0.6338	0.6296	0.6316
ResNet-50	0.7007	0.6609	0.6086	0.6336
MobileNetV2	0.6719	0.6838	0.5687	0.6206
EfficientNet-B0	0.7322	0.6586	0.6363	0.6472
ShuffleNetV2	0.7619	0.6237	0.6190	0.6213

**Table 3:** Comparison of classification results for ablation network models. (All experiments were conducted on the same dataset. The designation with an “*” indicates that the model has been pretrained.

	Accuracy	Precision	Recall	F1-Score
Single (global)	0.7923	0.7857	0.5840	0.6760
Single (local)	0.7643	0.7487	0.7463	0.7912
Dual (No pretrained)	0.7947	0.7862	0.7907	0.7882
Dual + Attention	0.8110	0.8113	0.8007	0.7951
Dual * (Pretrained)	0.8376	0.8213	0.8167	0.8032
DLRNet	0.8572	0.8557	0.8513	0.8562

**Table 4:** Wilcoxon signed-rank test for original predictions and model predictions.

Paired Variable	Median ± Standard Deviation (SD)			Z Value	*p* Value	Cohen’s d
	Pair 1	Pair 2	Paired Difference (Pair 1 − Pair 2)			
Physician Prediction vs. DLRNet Prediction	1.000 ± 0.819	1.000 ± 0.768	0.000 ± 0.626	6.275	0.000 ***	0.989

*** *p* < 0.001.

**Table 5:** Comparison with other State-of-the-Art methods on the same dataset.

Method	Accuracy	Precision	Recall	F1-Score
CNN [33]	0.7225	0.7223	0.7225	0.7217
VGG Net-D & Net-E [34]	0.7239	0.7264	0.7239	0.7219
Mobilenets [35]	0.7786	0.7749	0.7748	0.7706
ResNet (ILSVRC’15) [36]	0.7800	0.7867	0.7869	0.7842
DenseNet-BC [37]	0.7900	0.7914	0.7937	0.7957
GoogLeNet [38]	0.8186	0.8129	0.8193	0.8146
EfficientNet [39]	0.8329	0.8281	0.8324	0.8349
MobileNet-RVFL-CBA [40]	0.8110	0.8133	0.8013	0.8241
ResNet-ELM-CBA [41]	0.8061	0.8067	0.8091	0.8007
BN-AlexNet-ELM-CBA [42]	0.8225	0.8204	0.8213	0.8115
Deep transfer learning [43]	0.8005	0.8072	0.8008	0.8094
AlexNet + TL	0.8139	0.8227	0.8162	0.8127
ELNet [44]	0.8173	0.8145	0.8057	0.8060
Cross [45]	0.8157	0.8263	0.8197	0.8201
MedCLIP (image)	0.8219	0.8306	0.8134	0.8290
CLIP [46]	0.8328	0.8465	0.8390	0.8280
DLRNet	0.8572	0.8557	0.8513	0.8562

## Data Availability

The endoscopic dataset generated and analyzed during the current study is not publicly available due to the protocol of the Department of Obstetrics and Gynecology at Renji Hospital, but data are available from the corresponding author upon reasonable request.
